# Relationship between wettability of pulp fibers and tensile strength of paper during recycling

**DOI:** 10.1038/s41598-022-05514-2

**Published:** 2022-01-28

**Authors:** Hailan Jin, Ryota Kose, Nobushige Akada, Takayuki Okayama

**Affiliations:** 1 Key Laboratory of Bio-based Material Science & Technology (Northeast Forestry University) Ministry of Education, No. 26 Hexing Road, Harbin, 150040 China; 2 Division of Natural Resources and Eco-Materials, Institute of Agriculture, Tokyo University of Agriculture and Technology, 3-5-8 Saiwai-cho, Fuchu, Tokyo 183-8509 Japan; 3 Faculty of Agriculture, Tokyo University of Agriculture and Technology, 3-5-8 Saiwai-cho, Fuchu, Tokyo 183-8509 Japan; 4 State Key Laboratory of Biobased Material and Green Papermaking, Qilu University of Technology, Shandong Academy of Sciences, Jinan, 250353 China

**Keywords:** Engineering, Materials science

## Abstract

The wettability of the paper surface is greatly affected by the wettability of the pulp fibers. We conducted this study in order to understand the relationship between the wettability of a single fiber of recycled pulp and the strength of recycled paper, as well as the inter-fiber bonding strength. The contact angle was determined from a series of photographs of the pulp fiber and the water silhouettes at the point of contact. The contact line and profile history were continuously photographed in every 1 s after the initial contact. The recycled softwood kraft pulp fibers were clearly much less hydrophilic than the original fibers, regardless of whether the fibers had been bleached or not. The contact angle of the original chemi-thermomechanical pulp fiber was much higher than that of the original softwood bleached kraft pulp fiber. Furthermore, increased number of recycling decreased the contact angle of the chemi-thermomechanical pulp fiber. The Page equation was used to evaluate the strength contributions of single fiber and fiber–fiber bonding to tensile strength of paper. As a result, an increase in weakness factor of fiber–fiber bonding strength was obtained for the recycled softwood kraft pulp handsheet. On the other hand, the weakness factor of the original chemi-thermomechanical pulp handsheet decreased with recycling. In addition, the weakness factor of fiber–fiber bonding strength and the contact angles of the provided softwood bleached kraft pulp fibers bore a proportional relationship to each other.

## Introduction

Paper is said to be environmentally friendly as a natural recycling material and a sustainable recycling resource. The paper recycling system has been constructed as an advanced field of recycling. In particular in Japan, recycling of used paper has been promoted from the viewpoint of effective use of resources and solution of waste problems. The wastepaper recovery rate began a rapid and long-term rise as recovery efforts in recent years. The recovery rate in Japan had reached 79.5% in 2019^1^. The wastepaper utilization rate at 64.3% within Japan was trailing by 15.2% compared with the recovery rate. However, the ratio of wastepaper to the raw material of paper and paperboard in Japan is one of the world’s leading. Although Japan's waste paper recovery is at a fairly high level, there is a concern that the paper and paperboard recyclability will decline as the recovery rate rises. The utilization of recovered paper for high-grade paper such as printing and communication papers has been limited because using recovered paper tends to reduce the quality of these papers.

Mckee^2^ performed recycling of papermaking, drying, wetting and disintegration up to 6 times using softwood unbleached kraft pulp (SUKP). It was reported for the first time that physical properties of paper such as paper density, tensile strength, bursting strength, elongation, bending resistance and zero span tensile strength, decreased by recycle treatment but tearing strength and Taber stiffness increased when compared at the same freeness. Numerous studies have been conducted on the papermaking potential of recycled pulp fibers during the past decades^3–6^.

The cell wall of wood pulp fiber can be distinguished into a primary wall (P layer) and a secondary wall (S layer), and the S layer is further divided into an outer layer (S1), a middle layer (S2), and an inner layer (S3). Among them, the S2 layer occupies approximately 70–80% of the cell wall thickness and is the main cell wall. In the case of chemical pulp, since lignin and the like are removed in the process of pulping, voids are formed in the cell wall portion and the pulp becomes porous^7^. When the chemical pulp is repeatedly defibrated in water, dewatered on wet presses and dried on a paper dryer up to several times, not only are strength properties of the paper, such as tensile and bursting strengths significantly reduced, but also the micro-structure of the pulp fibers is damaged^8^. The recycling process causes morphological changes such as delamination and crack formation in cell wall of the pulp fibers^8,9^. The solute exclusion method has been devised by Stone and Scallan to elucidate the structure of pulp fiber cell walls in the presence of water^10,11^. The solute exclusion method utilizes the phenomenon that solute molecules invade pores in the cell wall according to the molecular diameter when the wet pulp is immersed in monosaccharides and dextran aqueous solutions having various molecular diameters. It was found by using the solute exclusion method that the pore volume in cell wall of the pulp fibers decreased with the number of recycling increased^10,12^. Stone and Scallan^13^ also noted significant decreases in the specific surface areas of once-dried bleached sulfite pulp by using nitrogen adsorption technique. It was confirmed that the decrease in the specific surface areas of the dried bleached sulfite pulp increased with an increase in drying temperature.

The decrease in the potential of recycled pulp fibers with respect to the strength of paper is largely due to changes in the cell wall structure of the fibers themselves. This change affects the swelling potential and conformability of the fibers in the papermaking process, and controls the refining characteristics^14,15^.

In the pulp fiber wall, the S2 layer repeatedly swells and shrinks due to recycling treatment, and microscopically forms a matrix structure with few fine pores. However, on the other hand, densification causes a twitching phenomenon in the matrix, and macroscopically, layered cracks are generated in the fiber wall, and cracks are also generated in the radial direction. Therefore, the inter-fiber bond formed by recycling is broken, and the strength of the paper sheet is also reduced^8,9^.

The so-called “hornification” of pulp fibers caused by recycling is the irreversible loss of fiber swelling, which is determined as water retention value. The irreversible hornification leads to remarkable reductions in fiber–fiber bonding. It occurs strongly in the fiber cell wall matrix of chemical pulp, but not so much in mechanical pulp^6^.

Cidir et al.^16^ also confirmed that as a result of papermaking using refined bleached sulfite pulp (BSP) and then recycling, the tensile strength, tearing strength and elongation of the paper decreased, but the opacity improved. Furthermore, by measuring and substituting the zero-span tensile strength, which is an index of single fiber strength, into the Page's equation^17^, it was evaluated that the decrease in paper tensile strength due to recycling depended on the decrease in fiber–fiber bonding strength. Howard et al.^18^ showed that there is few changed in both wet and dry zero-span tensile strength during recycling of various pulps.

Regarding the strength of paper such as tensile and bursting strengths, hydrogen bonds are considered to be the most important for the bonding strength of cellulose fibers in paper. The ability of cellulose fibers to form fiber–fiber bonds depends on the hydrophilicity of the fiber surface, that is, the ability to form hydrogen bonds based on it^19^. Since fiber–fiber bonds are formed between fibers interacting in water, wet adhesion influenced by the wetting of a fiber–fiber bonding and the strength of fiber network. It is important to gain a better understanding of the effect of the surface behavior of a single pulp fiber, because the fiber–fiber bonding strength is influenced by both the physicochemical properties of the pulp fiber surface and the contact area.

The evaluation of the paper surface wettability contributes to the control of various industrial processes. Young^20^ prototyped a contact angle measuring device using Wilhelmy's principle and determined the wettability of single pulp fibers. That is, after measuring the weight increase and the peripheral length of the fibers generated when the pulp fibers are suspended from a balance and immersed in a liquid, the contact angle is calculated using the following equation, and the wettability W is calculated by F/P.

1 $${\text{F }} = {\text{ P}}\gamma_{{{\text{LV}}}} \,{\text{cos }}\theta$$

where F is the tensile force acting on the solid rod immersed in the liquid, P is the peripheral length of the rod along the boundary line of the three phases, γ_LV_ is the surface tension of the liquid, θ is the contact angle. The contact angle of pulp fibers with water was reported to be 52° for Douglas fir unbleached kraft pulp (UKP) fibers and 43° for Aspen thermomechanical pulp (TMP) fibers. From the dynamic wetting test of wood pulp fiber by Wilhelmy method, unbleached neutral sulfite semi-chemical pulp (NSSCP) fibers have higher wettability than UKP and TMP fibers. The reasons for this were the degree of removal of lignin on the fiber surface, the presence of hemicellulose, other carbohydrates and extract components, and suggested the effect of the sulfone group introduced into lignin^20^.

Klungness^21^ modified Young's equipment to improve measurement accuracy and determined the contact angle of water to loblolly pine kraft pulp fibers with different lignin contents. As a result, it was confirmed that the contact angle with water increases due to the hydrophobic effect of lignin as the lignin content in the pulp fiber increases. Jacob et al.^22^ measured the contact angle of pulp fibers using liquids with different surface tensions and attempted to calculate the critical surface tension from the Zisman technique. Although the results showed significant variations in surface characteristics within a single fiber type, chemi-thermomechanical pulp (CTMP) fibers were more wettable compared with softwood and hardwood UKP fibers.

When the critical surface tension was measured by the Zisman technique using a film sheet prepared from each of the three main constituents of wood, it was estimated that a relatively large amount of lignin was distributed on the fiber surface in UKP^23,24^. On the other hand, in the case of TMP fibers, it depends on the temperature condition during the process because of the relationship with the softening temperature of lignin. Therefore, it was concluded that the wettability of pulp fibers depends on the chemical composition and structure of the fiber surface^20^. In general, chemical pulp fibers such as bleached kraft pulp (BKP) and UKP with low lignin content have been found to be more hydrophilic than high yield pulp fibers such as groundwood pulp (GP), refiner mechanical pulp (RMP) and TMP fibers with high lignin content^25^.

Berg^26^ evaluated the surface free energy, including the contribution of acid–base interactions, from the wetting measurements of pulp fibers. As a result, it was shown that the BKP fiber has a slightly larger dispersion force component γ_sd_ and a considerably smaller electron donor (base) parameter γ_s_—of the surface free energy than the CTMP fiber. In addition, Yoshinaga et al.^27,28^ devised a method of directly measuring the contact angle by continuously photographing the contact interface between the wood pulp fiber and the wet liquid using a stroboscope every 0.2 s. The dry and wet recycling treatment for pulp significantly increased the contact angle of water with respect to the pulp fibers, but tended to decrease the contact angle of water on the paper surface. In addition, Okayama et al.^29^ evaluated the change in surface free energy generated in pulp fibers by recycling, including the contribution of acid–base interaction, and found that γ_s_ hardly changed but γ_s_-decreased.

Although many studies on paper recycling have been conducted over the years, it is well known that recycled pulp fibers reduce the strength of paper, mainly tensile strength, compared to virgin fibers. The effect of repeated wetting and drying treatments on the reduction of bond-forming ability between pulp fibers has been mainly due to the loss of the bond region caused by the hornification of fibers. Therefore, it has been considered important to improve the fiber flexibility in order to regain the bond-forming ability of fibers. However, it is not easy for the recovery of the tensile strength of paper prepared from recycled fibers by refining to reach the level of undried fibers^15^. This indicates that the recovery of the bond-forming ability of dried fibers does not reach that of undried fibers.

Eastwood et al.^30^ examined the effects of additional refining of pulp, stock preparation by adding sizing agents, and papermaking with hand-made or machine-made paper on the physical properties of paper for each recycling of semi-bleached kraft pulp. It was clarified that the recycling process by preparing the hand-made paper has a significant decrease in the tensile strength of the paper and greatly reduces the hemicellulose content in the paper as compared with the machine-made paper using a white water circulation system. As a result, it was concluded that the decrease in paper strength due to recycling is caused not only by the decrease in swelling capacity of secondary fibers but also by the deterioration of the fiber surface condition. Seth and Page^31^ and Gurnagul et al.^32^ argued that the decrease in the tensile strength of paper during drying is affected not only by the relative bond area but also by the decrease in shear bond strength from the examination by the Page's equation. Therefore, in order to improve the strength characteristics of recycled paper, it is necessary to focus on increasing the bonding strength between fibers^32,33^.

The wettability of the pulp fiber surface is an important factor for the fiber–fiber bonding force, and it is considered that the improvement thereof increases the swelling property, flexibility and specific surface area of the fiber and leads to the strengthening of the fiber–fiber bonding force^34,35^. The wettability of the pulp fiber surface has a great influence on the swelling of the fiber and the liquid permeability of the paper, and the surface free energy of the pulp fiber can be calculated from the measurement of the contact angle of the liquid on the fiber surface. However, few studies have clarified the effect of the wettability of pulp fibers on the paper strength and fiber–fiber bonding of recycled fibers.

In this study, in order to investigate the effect of the physicochemical properties of recycled pulp fibers on the paper strength, recycled pulps with different lignin contents prepared from softwood UKP and CTMP was examined from the change in the fiber–fiber bonding force based on the Page equation.

## Materials and methods

### Pulp samples

Commercially available softwood bleached kraft pulp (SBKP), softwood unbleached kraft pulp (SUKP) and chemi-thermomechanical pulp (CTMP) were used (Table 1). SUKP and SBKP were manufactured from mixed chips of Japanese larch, Douglas fir and slash pine, and CTMP was manufactured from Todo fir.

**Table 1:** Klason lignin content of softwood pulp samples.

	Sample	Softwood pulp samples	Delignification treatment	Klason lignin (%)
SKP	SBKP	Bleached kraft pulp	ECF-bleached	0.0
	SUKP	Unbleached kraft pulp	–	5.7
	DSUKP	Unbleached kraft pulp	Delignified	3.5
	CTMP	Chemi-thermomechanical pulp	–	37.9

### Bleaching of pulp samples

Chlorine dioxide bleaching treatment was performed on SUKP to prepare chlorine dioxide bleached softwood kraft pulp (DSUKP). For chlorine dioxide bleaching, 20 g of pulp was placed in a Lamizip. After 8 g of sodium chlorite powder was added, 4 ml of acetic acid were poured into the Lamizip together with 1200 ml of water. The zipper of the Lamizip was closed to prevent chlorine dioxide gas from escaping to the outside, and the Lamizip was placed in a hot water bath at 70–80 °C. After reacting for 1 h, 8 g of sodium chlorite and 4 ml of acetic acid were added. SUKP repeated this process twice. After completion of the reaction, the contents of the Lamizip were suction-filtered on a Büchner funnel, washed until acetic acid was exhausted, and finally washed with hot water. Lignin content was measured for all pulp samples according to TAPPI TEST METHOD T222. The results are shown in Table 1.

### Recycling of pulp samples and measurement of physical properties of paper

Pulp samples were beaten using a Valley beater according to ISO 5264- at a pulp concentration of 2% until a freshness of 400–450 mL CSF. In order to prepare recycled pulps, handsheets were prepared from each beaten pulp sample based on ISO 5269-1. The handsheets were used as a sample (hereinafter abbreviated as R0) that was recycled 0 times. On the other hand, a part of the handsheets were dried at 80 °C for 24 h in a forced air circulation oven after the pressing. Further, after immersing the dried handsheets into water for 1 h, the handsheet was sufficiently defibrated by a disintegrator to prepare the handsheet again. This was used as a sample for one recycle (hereinafter abbreviated as R1). In this procedure, wetting and drying were repeated up to 3 times to prepare recycled pulp handsheet samples (hereinafter abbreviated as R3). The prepared test handsheet was adjusted for humidity all day and night under environmental conditions of 23 °C and 50% RH, and then the sheet density and tensile strength were measured according to ISO 5270, and the zero-span tensile strength was measured according to ISO 15361.

### Measurement of dynamic contact angle of pulp fiber

**Figure 1:**
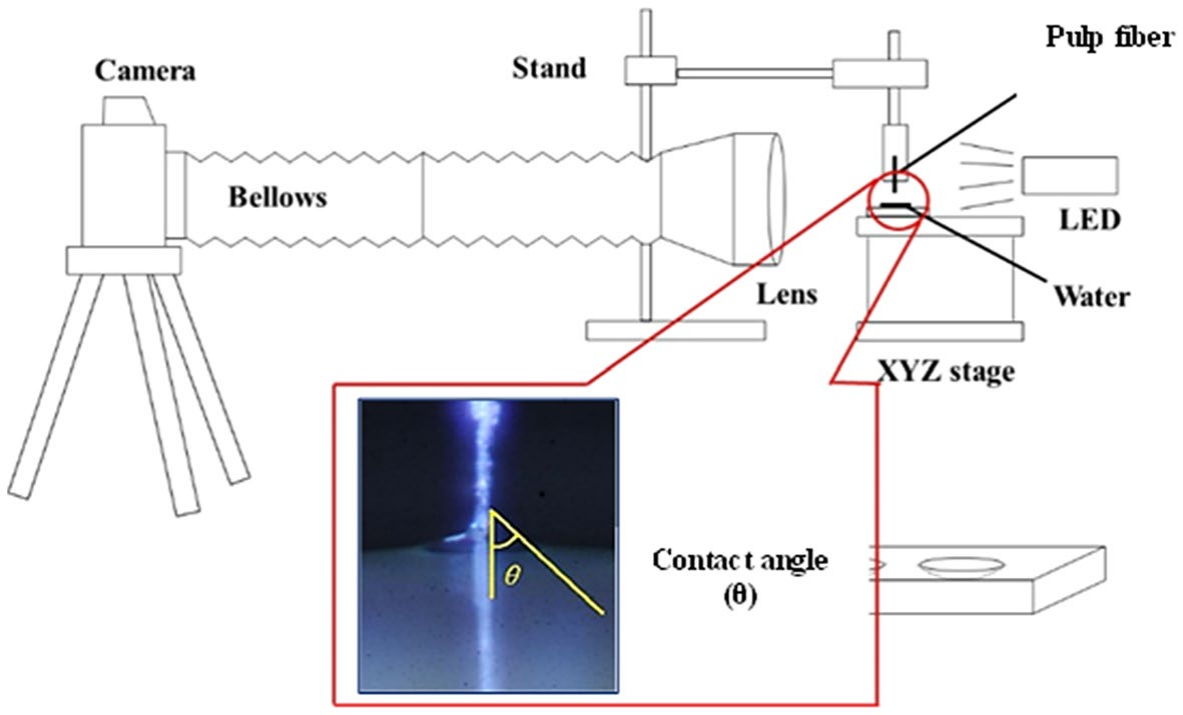
Schematic diagram of measuring water contact angle between single pulp fiber and water.

The optical technique to measure directly the contact angle of a water drop against a single pulp fiber was used for characterizing fiber-liquid interactions. A system consisting of a single-lens reflex camera (Nikon D3), a 24 mm wide-angle lens (Nikon), a bellows attachment (Nikon MD-4), an extension bellows attachment (Nikon BP-6E) was used for the measurement (Fig. 1). A xyz stage (Chuo Precision Industrial) was also used to hold a pulp fiber. The 24 mm wide-angle lens mounted to the bellows attachment was attached to the camera. The extension bellows attachment connected in series to increase the shooting magnification.

**Figure 2:**
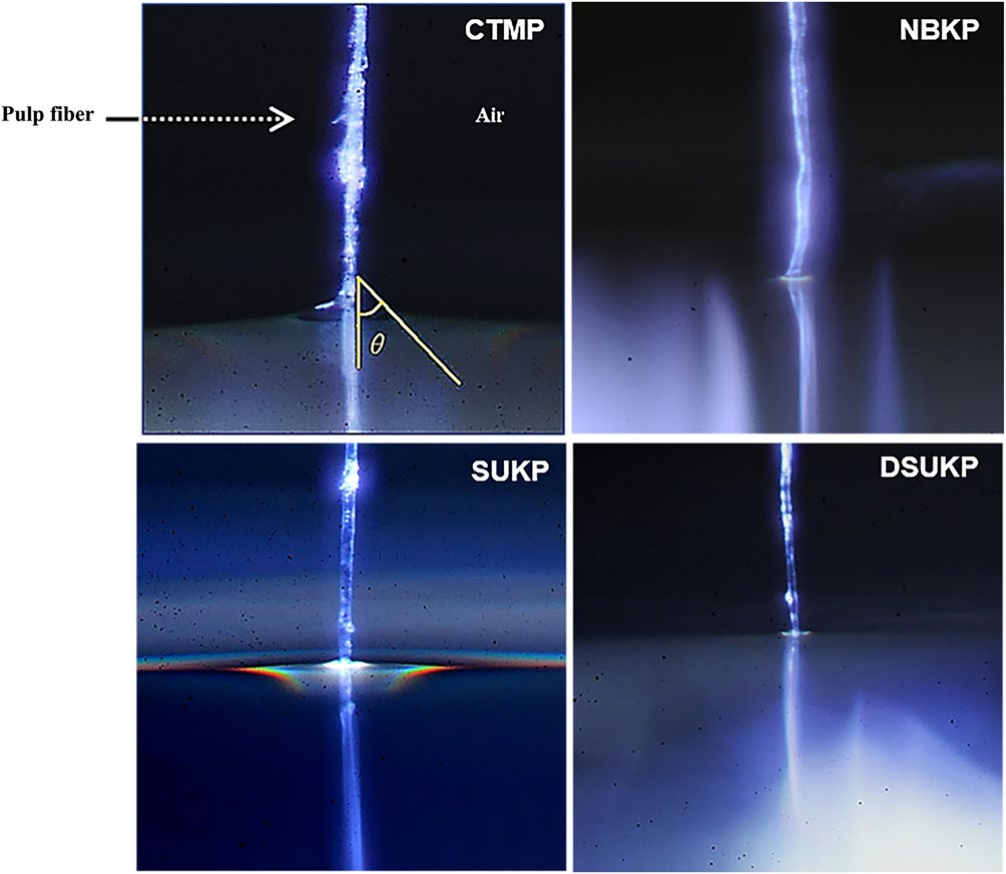
Water contact angle of a single pulp fiber.

**Figure 3:**
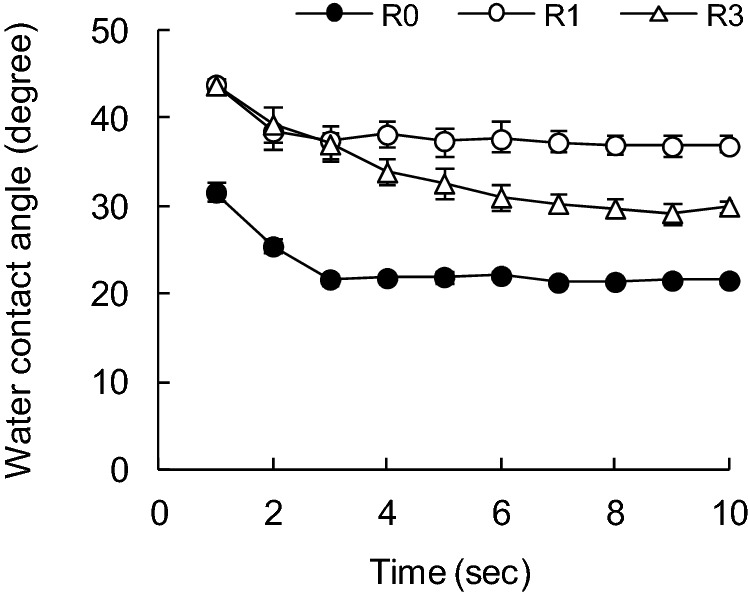
Effect of recycling on water contact angle of SBKP fibers with time variations.

**Figure 4:**
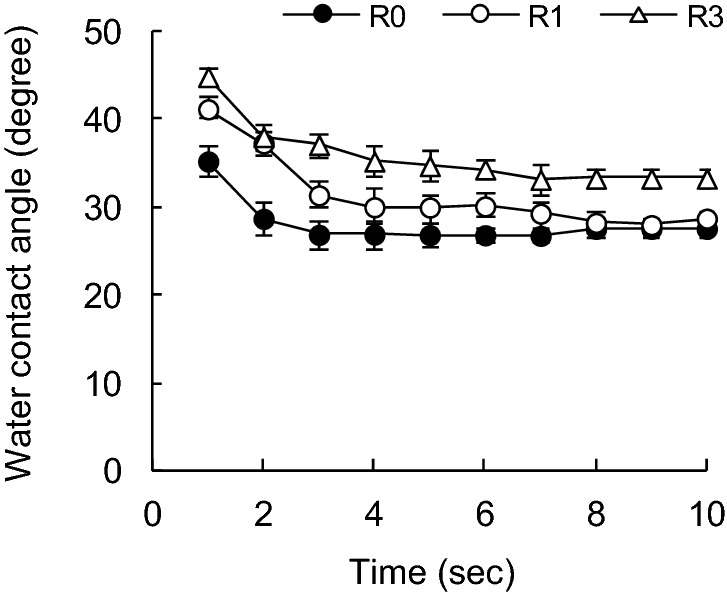
Effect of recycling on water contact angle of SUKP fibers with time variations.

**Figure 5:**
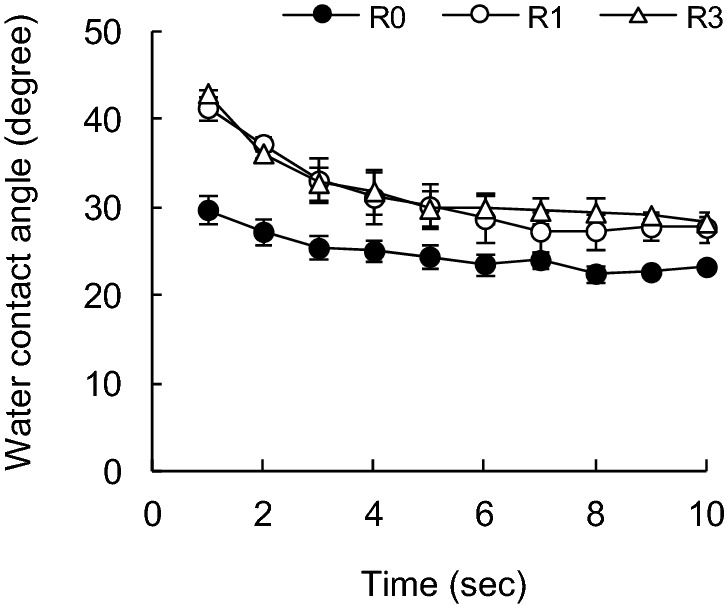
Effect of recycling on water contact angle of DSUKP fibers with time variations.

**Figure 6:**
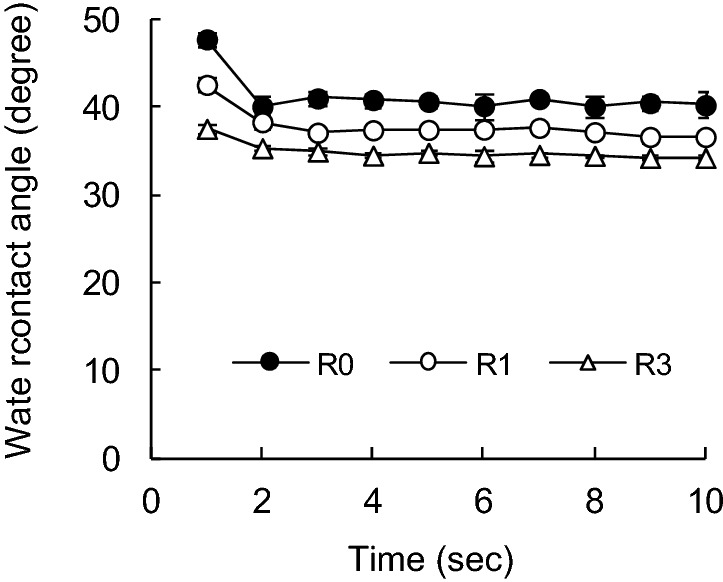
Effect of recycling on water contact angle of CTMP fibers with time variations.

The handsheet was carefully torn so as not to break the inter-fiber bonds in the paper sheet, and the surrounding fibers were excised, leaving one pulp fiber in the torn handsheet cross-section^36^. Pulp fibers were selected from relatively straight samples and measured 20 times per sample. After fixing the tip of the pulp fiber downward, the height of the tip of the pulp fiber was made constant. Pure water was put onto a slide glass in which a liquid reservoir was placed on the z-direction lift, and the z-direction lift was raised to make the tip of the pulp fiber pure water. The altitude at which the z-direction lift was raised was also constant, and the distance at which the pulp fibers were inserted into the water was the same^27,29^. After the tip of the pulp fiber came into contact with water, the area around the pulp fiber was continuously magnified and photographed every second for 10 s or more (Fig. 2). The P values between all of the data were all 0.05 or less, thus indicating a significant difference. The starting point of measurement (0 s) was defined as the point where fiber insertion could be confirmed from the continuously captured images. When the pulp fiber is in contact with water, the contact surface is quickly wetted and absorbed water. At this time, the contact angle changes to a certain extent, until the contact angle becomes stable when it is saturated (Figs. 3, 4, 5, 6).

## Results and discussion

### Effect of recycling treatment on surface wettability of pulp fibers

Figures 3, 4, 5 and 6 show the changes over time in the contact angle of water in various recycled pulp fibers. In the case of SBKP, the contact angle increased with the number of recycling increased (Fig. 3). The contact angle at 1 s after water comes into contact with the pulp fiber was 32° for the pulp fiber (R0) before recycling, while it was 44° after the first recycling (R1). However, the contact angle was 44° even after 3 times of recycling (R3). There was little difference in the contact angle at 1–3 s after the contact, even if the number of recycling treatments increased. Therefore, it was suggested that the first recycling treatment out of the three recycling treatments had a particularly large effect on the wettability of pulp fibers. The increase in contact angle due to the recycling process of pulp fiber can be caused by both the hornification of the pulp fiber surface shown in the decrease in pore volume in the fiber cell wall^12,15^, and the decrease in surface bonding potential^30,32,33^.

On the other hand, Wistara et al.^37^ measured the contact angle of water using the Wilhelmy method to evaluate the surface properties of recycled pulp fibers, and found that the recycling treatment reduced the contact angle of bleached kraft pulp fibers. Regarding the change in wettability on the pulp fiber surface due to recycling, our results tended to differ from their results because of the difference in the treatments of the recycling process. That is, in the test of Wistara et al., the pulp was beaten after being repeatedly defibrated in water, dewatered and dried in the recycling process, but in our experiment, the fact that the pulp was not beaten after the recycling process had an effect. It is presumed that the surface of the pulp fiber after the recycling treatment was roughened by the subsequent beating treatment, the chemical composition of the fiber surface was changed, the hydrophilic surface was exposed, and as a result, the wettability was improved.

The contact angle of water on the fiber surface before and after recycling (R0 and R1) has been stabilized at 4 s after the contact of water with the SBKP fiber. However, the water contact angle for the fibers after the three recycling treatments (R3) was lower after 4 s of contact compared to the fibers after the first recycling treatment (R1). The contact angle of water showed a tendency to gradually decrease as the number of recycling increased after 4 s of contact. This can be related to the appearance of radial cracks in the S2 layer of the fiber cell wall confirmed by TEM observation, when the SBKP fiber is recycled 3 times or more^8,9,15^. That is, the reason why the contact angle of water on the surface of SBKP fiber recycled 3 times or more gradually decreases is that as the contact time between the fiber surface and water becomes longer, water is penetrated into the cracks generated in the fiber cell wall of the recycled pulp. As a result, it is estimated that the water contact angle has decreased.

As with the SBKP, the contact angle of water with respect to the SUKP fibers increases as the number of recycling increases (Fig. 4). The contact angle of the SUKP fiber after 1 s contact with water was 35°, which was slightly higher than that of the SBKP fiber. The SUKP fiber contains 5.7% of lignin (Table 1), and the change in the contact angle of water is influenced by the residual hydrophobic lignin. It would be caused that the surface of cellulosic fibers becomes hydrophobic again due to the redistribution of olefinic substances derived from wood materials, in addition to hornification of pulp fibers and cleavage due to hydrolysis of covalent bonds of cellulose chains during recycling^38^. In unbleached chemical pulp and mechanical pulp, lignin remains in the pulp, and the presence of triglyceride fat, fatty acid, resin acid and unsaponifiable matter has been confirmed, and it is possible to defibrate pulp fibers and heat-dry paper in the papermaking process. It is said that storage causes a self-sizing phenomenon in which these low-surface free-energy substances migrate and spread to the surface of pulp fibers^34,39,40^.

Hodgson et al.^25^ determined the water contact angle of pulp fibers using the Wilhelmy method after heat-treating Douglas fir kraft pulp at 105 °C for 16 h, and found that this heat treatment increased the contact angle. Therefore, it is shown that the wettability of the pulp fiber is suppressed by the self-sizing effect.

Therefore, the contact angle of the SUKP pulp fibers after the first recycling increased to 41°, and after third recycling, the contact angle increased to 45°, showing a tendency for the contact angle to increase with the number of recycling. It is considered that this is due to the effect of self-sizing. In addition, the contact angle of water with respect to the SUKP fiber after three recycling treatments was larger than that of the SBKP regardless of the passage of time. When the SUKP was delignified (DSUKP), the recycling treatment increased the contact angle of the pulp fibers (Fig. 5). The contact angle of the DSUKP pulp fiber before recycling is 30°, which is lower than that of the SUKP. The lignin content of the DSUKP pulp fiber was 3.5%, when the SUKP was delignified. It is considered that as the hydrophobicity of the pulp fiber was reduced, the contact angle was lowered. In addition, it was confirmed that the contact angle of the pulp fiber increased to 41° after the first recycling treatment and 43° after three recycling treatments, but it was somewhat lower than the SUKP.

**Figure 7:**
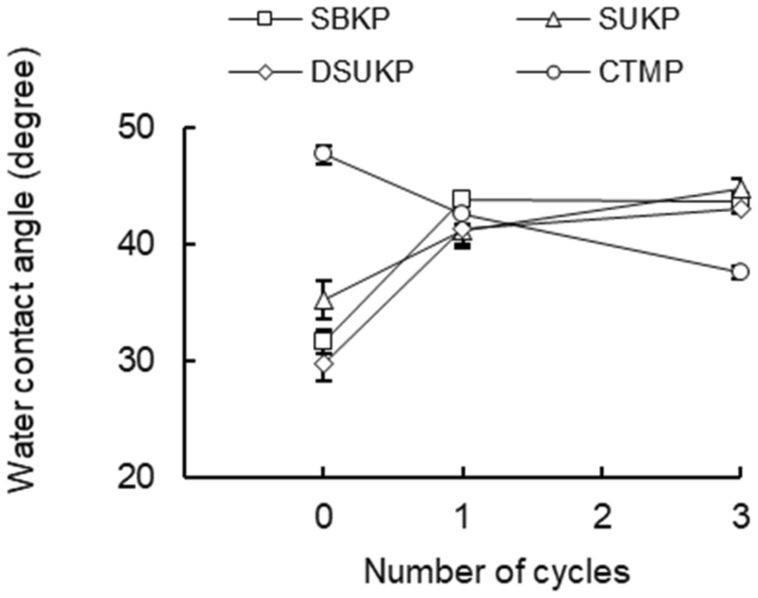
Effect of recycling on contact angle of pulp fibers immersed in water after contact 1 s.

**Figure 8:**
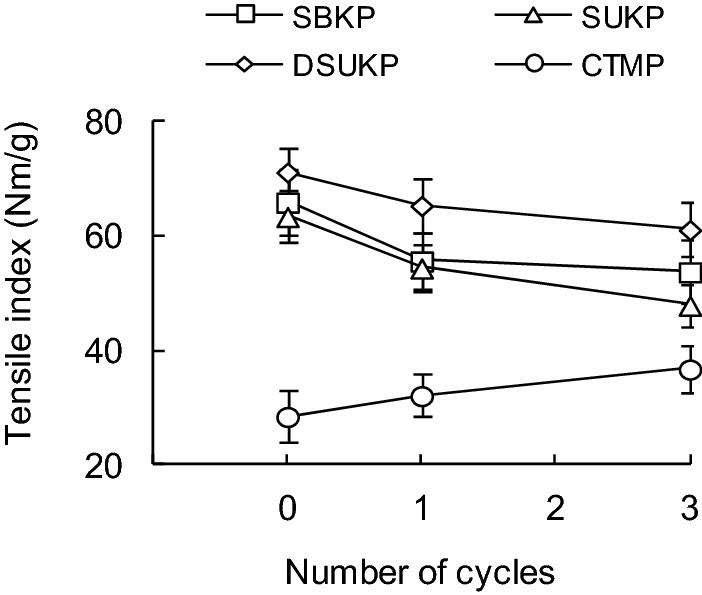
Effect of recycling on tensile strength of handsheets prepared from various pulps.

In the case of the CTMP, the contact angle of pulp fibers tended to decrease after the recycling treatment (Fig. 6), showing a tendency different from that of softwood pulp fibers (SKP) whose water contact angle increased due to recycling. The contact angle before the recycling treatment was 48°, and the contact angle decreased to 43° after the first recycling treatment and 38° after three recycling treatments. It is presumed that the decrease in contact angle with the increase in the number of recycling is due to the decrease in the content of lignin and wood-derived resin during the recycling process. A slight increase in the tensile strength of the CTMP sheet due to the recycling process (Fig. 8) also supports this possibility. The contact angle of CTMP is higher than that of softwood pulp fiber, and it can be judged that the effect of hydrophobicity of lignin and resin increases the contact angle with water^21^. Figure 7 shows the effect of recycling treatment on the contact angle of pulp fibers at 1 s after contact with water. It was clarified that the contact angle of SBKP and SUKP fibers tends to increase as the number of recycling treatments increases, but the contact angle of CTMP fibers decreases as the number of recycling increases.

### Effect of recycling treatment on tensile strength of paper sheets

Figure 8 shows the changes in the tensile strength of handsheets prepared from each pulp sample by recycling. As the number of recycling treatments increased, the tensile strength of the handsheets prepared from the SKP pulps tended to decrease. In the case of the SBKP, the tensile index of handsheets gradually decreased from 65.9 N m/g in R0 to 55.7 N m/g in R1 and 53.8 N m/g in R3 due to the recycling process. There was a tendency that the tensile index of the SUKP handsheets is lower than that of the SBKP, which decreases from 63.4 N m/g in R0 to 54.5 N m/g in R1 and 48.0 N m/g in R3 due to recycling. It was revealed that delignification (DSUKP) treatment using sodium chlorite and acetic acid improves the tensile strength of handsheets before recycling. In addition, the tensile strength of DSUKP handsheets was higher than that of the SUKP regardless of the presence or absence of recycling treatment. Since the presence of lignin interferes with the formation of interfiber bonds in the handsheets, the delignification treatment could improve the tensile strength of the handsheets. The tensile index of DSUKP handsheets decreased from 71.2 N m/g before recycling (R0) to 65.4 N m/g for R1 and 61.1 N m/g for R3 as the number of recycling increased.

On the other hand, in the case of the CTMP in which components other than cellulose such as lignin remain, the tensile index of handsheets increases from 28.6 N m/g in R0 to 32.2 N m/g in R1 and 36.8 N m/g in R3 as the number of recycling increases. R3 showed a tendency to increase slightly to 36.8 N m/g. This is partly due to the fact that in experiments conducted by Howard et al.^18^ using groundwood pulp (GP) and CTMP, recycling treatment tends to increase strengths such as tensile strength and burst strength of papers produced from mechanical pulp. It is considered that lignin was removed by washing in the recycling process and the inter-fiber bonding between pulp fibers was strengthened in the papermaking process of defibration in water, dewatering and drying, which contributed to the improvement of tensile strength.

Nazhad^25^ showed in experiments on the recycling process of CTMP that the fiber coarseness of CTMP was reduced and the fiber flexibility was added regardless of the degree of delignification treatment with sodium chlorite. Furthermore, it has been reported that in CTMP that has been subjected to the delignification treatment, the strength of the handsheets decreases due to the increase in the number of recycling, regardless of the amount of lignin content. On the other hand, in the CTMP experiment of this study, it was confirmed that the tensile strength of the handsheets tends to increase or relatively little change as the number of recycling increases. On the other hand, in the CTMP experiment of this study, it was confirmed that the tensile strength of handsheets tends to increase or relatively little change as the number of recycling increases. The effect of the recycling treatment on the tensile strength of the CTMP handsheets obtained in this study is different from that of SUKP and DSUKP because the wettability on the surface of the CTMP fiber improved and the fiber–fiber bond strength increased as the number of recycling increased.

### Effect of recycling treatment on fiber–fiber bond strength in paper sheets

To clarify the factors that influence the tensile strength of paper sheets, the Page equation^17,31^ has been proposed. Furthermore, Cildir et al.^16^ applied the Page equation below to calculate the fiber–fiber bond strength from the viewpoint that the resistance to fracture consists of two comparable resistances. It was presumed that the decrease in tensile strength was mainly the decrease in the fiber–fiber bond strength.

2 $${1}/{\text{T }} = { 1}/{\text{F }} + { 1}/{\text{B}}$$

3 $${\text{F }} = {\text{ 8Z}}/{9}$$

where T: tensile index of sheet, F: fiber strength index of sheet, Z: zero-span tensile index of sheet, B: bond strength index of sheet.

**Figure 9:**
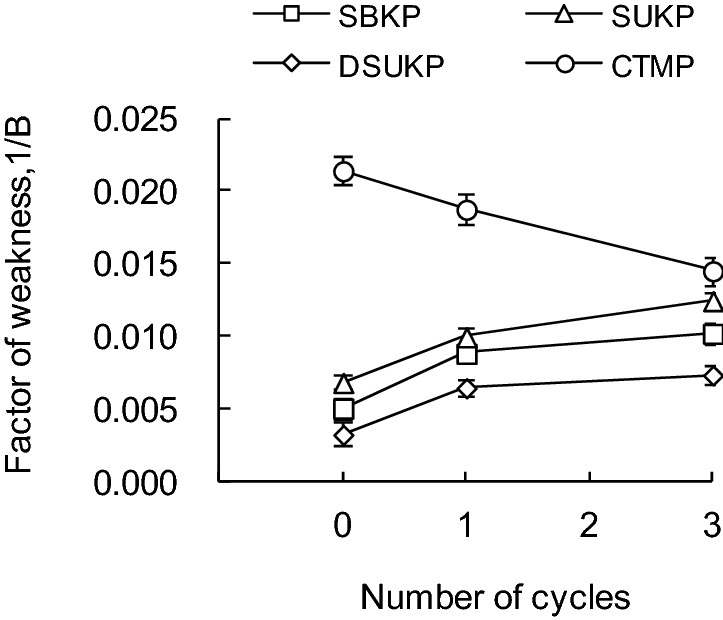
Changes in factor of weakness inter-fiber bond strength during recycling.

Using Eqs. (2) and (3), it should be possible to calculate the fiber–fiber bond strength index of sheet, B, if the tensile strength of sheet, T and the zero-span tensile index, Z are measured. Figure 9 shows the effect of the number of recycling treatments on the fiber–fiber bond strength index. As the number of recycling treatments increased, the factor of fiber–fiber bond weakness (1/B) of all SKP paper sheets tended to increase, and it became clear that the fiber–fiber bond strength became weaker. In addition, 1/B of DSUKP was lower than that of SUKP. By applying the delignification treatment, the lignin content in the paper sheet is reduced and the formation of fiber–fiber bonds is promoted. Comparing 1/B of SBKP and DSUKP, despite the fact that the DSUKP had a smaller of 1/B and a higher lignin content in pulp (Table 1), the result was obtained that the fiber–fiber bond strength of DSUKP was higher. On the other hand, in the CTMP paper sheet, 1/B decreased with the number of recycling, which means that the fiber–fiber bond strength in the paper sheet was improved.

### Effect of surface wettability of wood pulp fibers on fiber–fiber bonding potential

**Figure 10:**
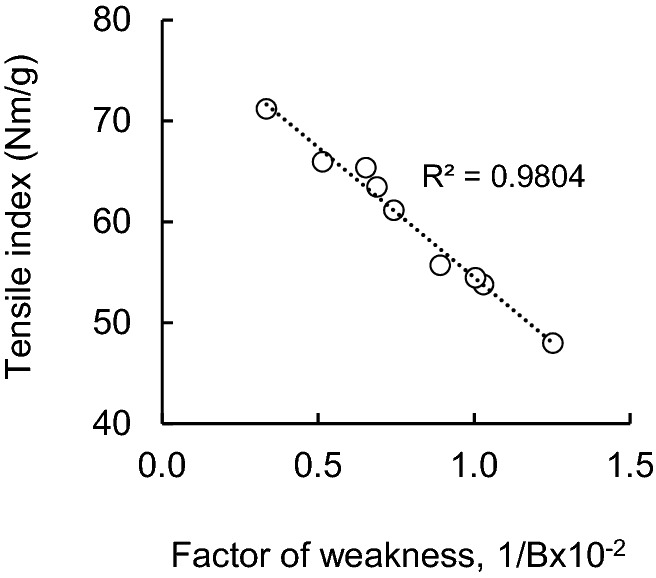
Factor of bonding weakness, 1/B versus tensile index of SKP handsheets.

The tensile strength of the paper sheet, the wettability of the pulp fiber, and the change in the bond strength between the pulp fibers in the paper sheet were evaluated by the recycling experiment of the SKP pulp fiber, and the correlation was examined. When all the values were evaluated for each sample used in this study, the correlation between the tensile strength of the sheet and the fiber–fiber bond weakness index was R^2^ = 0.98 (Fig. 10). The *p* value of the correlation coefficient was 0.000, which was judged to be significant at the 5% significance level. Therefore, it was found that the correlation between the tensile strength of the sheet and the fiber–fiber bond strength is considerably high.

**Figure 11:**
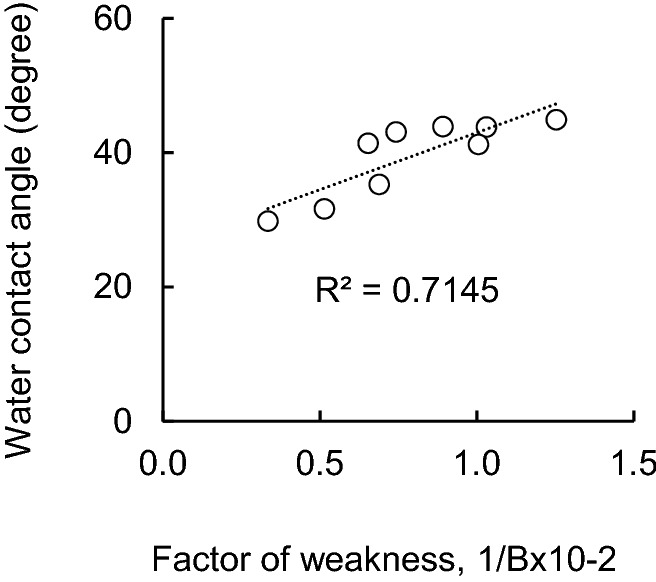
Factor of bonding weakness, 1/B versus water contact angle after contact 1 s (SKP).

When the relationship between 1/B and the contact angle of water with respect to the pulp fiber in the SKP sample was examined, R^2^ = 0.71 was obtained (Fig. 11). In this case, the *p* value was 0.004, which was significant when the *t* test was performed (*p* < 0.05). It was found that it was not uncorrelated, and it was confirmed that the correlation between the two factors was relatively high. Therefore, it was clarified that the better the wettability of the pulp fibers, the easier it is to form fiber–fiber bonds and the greater the tensile strength of the paper sheet. In addition, it was speculated that the main cause of the decrease in the tensile strength of the paper sheet due to recycling was the decrease in the fiber–fiber bond strength of the paper sheet, and that the decrease in the fiber–fiber bond strength was related to the decrease in the wettability of the pulp fibers.

## Conclusions

Pulp samples prepared from softwood bleached kraft pulp (SBKP), softwood unbleached kraft pulp (SUKP) with different lignin contents and chemi-thermomechanical pulp (CTMP) are subjected to one or three repeated wet and dry treatments. The contact angle of water with respect to each pulp fiber over time was measured. Furthermore, the effect of the wettability of pulp fibers on the tensile strength of the paper sheet was evaluated from the change in the fiber–fiber bond strength based on the Page equation.

1. In the case of the SBKP, repeated dry and wet recycling treatment increased the contact angle of water with fibers and reduced wettability. The pulp fiber (R3) that had been recycled three times maintained a contact angle similar to that of R1 within 1–3 s after contact. After 4 s of contact, it was smaller than the contact angle of the pulp fibers (R1) that had been recycled once. On the other hand, the CTMP fiber showed a different behavior from the softwood kraft pulp fiber (SKP) because the contact angle with water decreased as the number of recycling increased.
2. The tensile strength of softwood kraft pulp (SKP) paper sheets, including paper sheets prepared from bleached, unbleached and delignified pulp, tended to decrease as the number of recycles increased. On the other hand, in the paper sheet prepared from CTMP, the tensile strength tends to increase slightly as the number of recycling increases, suggesting that the fiber–fiber bond may be strengthened.
3. By measuring the tensile strength and zero-span tensile strength of a paper sheet prepared from recycled softwood kraft pulp (SKP) fibers and applying it to the Page equation, the change in the contact angle of water with respect to the pulp fiber over time can be determined.

The effect of the wettability of pulp fibers on the tensile strength of paper, especially the fiber–fiber bond strength, was evaluated. As a result, the tensile strength of the paper sheet from the recycled SKP pulp fibers decreased with the increase of the factor of fiber–fiber bond weakness index (1/B). Furthermore, it was clarified that the wettability of pulp fibers is improved and 1/B is decreased. Therefore, the decrease in the tensile strength and fiber–fiber bond strength of the paper sheet due to the pulp recycling process could be associated with the decrease in the wettability of the pulp fiber.
